# Lack of cytomegalovirus (CMV)-specific cell-mediated immune response using QuantiFERON-CMV assay in CMV-seropositive healthy volunteers: fact not artifact

**DOI:** 10.1038/s41598-020-64133-x

**Published:** 2020-04-28

**Authors:** Jorge Valle-Arroyo, Rocío Aguado, Aurora Páez-Vega, Ana B. Pérez, Rafael González, Gema Fornés, Julián Torre-Cisneros, Sara Cantisán

**Affiliations:** 1 Instituto Maimónides de Investigación Biomédica de Córdoba (IMIBIC)/Reina Sofia University Hospital/University of Cordoba, Cordoba, Spain; 2 Department of Microbiology, Reina Sofía Hospital, Cordoba, Spain; 3 Department of Immunology, Reina Sofia Hospital, Cordoba, Spain; 4 Blood Transfusion Center and Tissue and Cells Establishment, Cordoba, Spain; 5 Infectious Diseases Unit. Reina Sofía Hospital, Cordoba, Spain

**Keywords:** Medical research, Risk factors

## Abstract

The QuantiFERON-CMV (QF) assay measures cell-mediated immunity against cytomegalovirus (CMV-CMI), which is particularly useful in individuals susceptible to CMV infection such as transplant patients. A positive QF result identifies patients that are better protected against CMV infection. However, the significance of a negative QF result in CMV-seropositive individuals needs to be clarified. CMV-CMI was analyzed in healthy subjects using the QF assay, and, in parallel, the Flow-cytometric Assay of Specific Cell-mediated Immune response in Activated whole blood (FASCIA). FASCIA assay measures T-cell proliferation using CMV lysate as stimulus whereas QF assay use a mix of peptides. A total of 93 healthy volunteers were enrolled, and 13/71 CMV-seropositive individuals (18.3%) showed humoral/cellular discordance using QF assay (CMV+ QF−). Interestingly, with FASCIA assay CD4+ and CD8+ T-cell proliferations were lower in CMV+ QF− than in CMV+ QF+ individuals. Furthermore, CMV+ QF− volunteers had a lower level of anti-CMV IgG than CMV+ QF+ subjects. Discordant CMV+ QF− volunteers can be defined as low responder individuals since they show lower CMV-specific humoral and cellular immune responses in comparison to CMV+ QF+ individuals. Immune discordance shows the high heterogeneity of immunity to CMV in healthy subjects.

## Introduction

In the last years, a variety of assays have been developed to measure cell-mediated immunity against cytomegalovirus (CMV-CMI), where the basic principle is the CMV-specific stimulation of T cells for 6–24 hours in cell culture^1,2^. These techniques have been shown to be particularly useful in individuals such as transplant patients who are susceptible to CMV infection, since they identify who is better protected against CMV infection after transplantation, as has been reported in international guidelines on the management of CMV in solid organ or stem cell transplantation^3,4^. Specifically, the detection of CMV-CMI at pretransplant or posttransplant using QuantiFERON-CMV (QF), ELISpot or intracellular cytokine staining has been associated with a lower risk of CMV infection, not only in observational studies^5–9^.

Although most individuals show an agreement between CMV-serostatus and CMV-CMI, some of them have a discordance. Therefore, there are CMV-seropositive individuals without CMV-CMI, as well as CMV-seronegative individuals with protective CMV-CMI. Discordant individuals have been reported in both transplant patients and healthy virus carriers^8–12^.

The QuantiFERON-CMV is an *in vitro* assay that measures CMV-CMI by quantifying IFNG released by CD8+ T cells after stimulation with a pool of HLA-restricted CMV peptides^13^. In some observational studies carried out in our group in solid organ transplant patients we found that 20–25% of CMV-seropositive transplant candidates lacked CMV-CMI response using the QF assay, and they showed a higher risk of post-transplant CMV infection^9,14^.

However, the explanation for a negative QF assay result in CMV-seropositive individuals is controversial. Some authors argue that negative results might be related to the inability of certain individuals to recognize the peptides of the QF assay^15^. To clarify this point, we analyzed CMV-CMI response with QF assay, and, in parallel, with the Flow-cytometric Assay of Specific Cell-mediated Immune response in Activated whole blood (FASCIA), which measures the lymphocyte proliferative response after stimulation with CMV lysate^16^.

The aim of this work is to evaluate whether CMV-seropositive healthy individuals with a negative QF result show an impaired proliferative response against CMV lysate or this humoral/cellular discordance in CMV-seropositive individuals is an artifact of the QF assay related to the type of stimulus.

## Results

### Demographic characteristics of study subjects

A total of 93 healthy participants were enrolled in the study. Some demographic characteristics of the volunteers are shown in Table 1. Seventy-one individuals (76.3%) were CMV-seropositive.

**Table 1:** Demographic characteristics of the study population.

Characteristics	All (n = 93)
Age, mean (SD)	42.8 (12.0)
Gender, n (%)	
Female	26 (28.0)
Male	67 (72.0)
CMV-serostatus, n (%)	
CMV−	22 (23.7)
CMV+	71 (76.3)

SD, standard deviation; CMV−, CMV−seronegative, CMV+; CMV−seropositive.

### Analysis of humoral and cellular discordance

We first analyzed CMV-CMI response in all the subjects using the QF assay. Thirty (32.3%) individuals were QF− and 63 (67.7%) were QF+. No candidate had an indeterminate QF result.

In order to obtain the frequency of individuals with humoral/cellular discordance, we determined the agreement between serology and CMV cellular immunity. We found that 17 out of 22 (77.3%) CMV-seronegative volunteers were QF−. Of the CMV-seropositive individuals, 58 out of 71 (81.7%) were QF+. Therefore, 18 healthy subjects (5 CMV-seronegative and 13 CMV-seropositive) (19.3%) showed humoral/cellular discordance.

### Differences in proliferative response among healthy volunteers with and without humoral/cellular discordance: FASCIA assay

**Figure 1:**
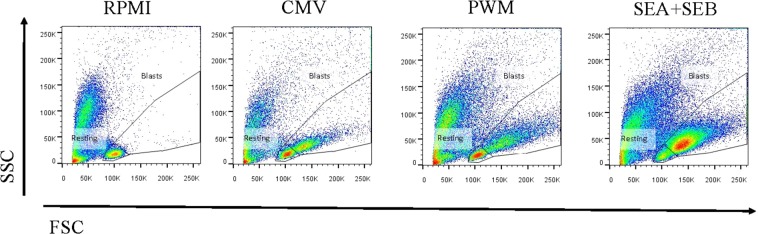
Analysis strategy for the evaluation of proliferative responses of lymphocytes in FASCIA assay by flow cytometry. Proliferative responses against CMV lysate, PWM, SEA + SEB and medium (RPMI) are shown. Resting and proliferating lymphocytes (blasts) are shown on a Forward Scatter (FSC) versus Side Scatter (SSC) dot plot.

To investigate whether the volunteers with and without humoral/cellular discordance exhibited differences in proliferative capacity, we compared the CD4+ and CD8+ T-cell proliferation between the two subgroups of CMV-seropositive (CMV+ QF+ and CMV+ QF−) and CMV-seronegative (CMV− QF− and CMV− QF+) individuals. A representative plot of the proliferative response against antigens and medium is shown in Fig. 1.

**Figure 2:**
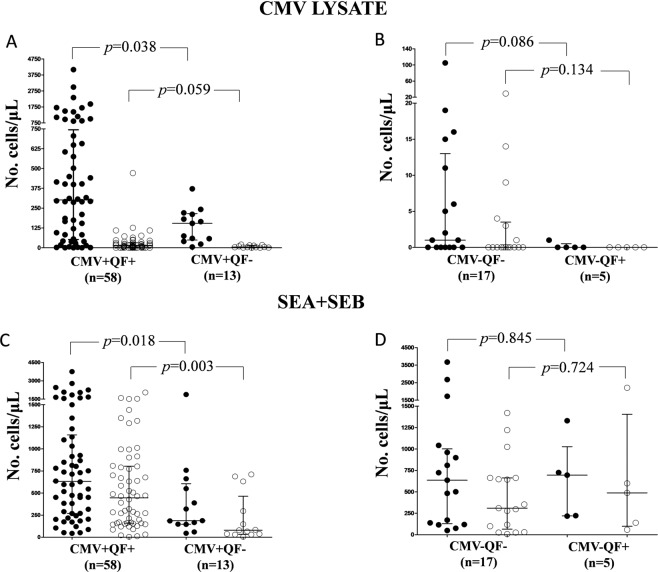
Comparative analysis of lymphocyte proliferation in discordant and non-discordant CMV+ and CMV− healthy volunteers. Proliferation of CD4+ and CD8+ T cells after stimulation with CMV lysate (**A,B**) and SEA + SEB (**C,D**). Horizontal lines represent median values. The interquartile range is also shown. Mann-Whitney test was used. Solid circles represent the CD4+ T-cells; white circles represent the CD8+ T-cells.

We observed significant differences between the two groups of CMV-seropositive subjects after stimulation with CMV lysate (Fig. 2A). Specifically, CD4+ T-cell proliferation was lower in CMV+ QF− than in CMV+ QF+ individuals (154.0 cells/μL vs. 301.5 cells/μL; *p* = 0.038). Regarding CD8+ T cells, we also found a tendency toward a lower proliferation in CMV+ QF− than in CMV+ QF+ subjects (4.0 cells/μL vs. 12.5 cells/μL; *p* = 0.059). No significant differences were detected in the group of CMV-seronegative individuals (Fig. 2B).

As regards the stimulation with superantigen (SEA + SEB), we also found significant differences in blast formation within the CMV-seropositive subjects (Fig. 2C). Proliferation of both CD4+ T cells (median 188.0 cells/μL vs. 631.5 cells/μL; *p* = 0.018) and CD8+ T cells (median 80.0 cells/μL vs. 446.0 cells/μL; *p* = 0.003) was lower in CMV+ QF− than in CMV+ QF+ subjects. There were no statistically significant differences in CMV-seronegative individuals (Fig. 2D).

We carried out the same comparative analysis with the B-lymphocyte proliferation after PWM stimulation. We did not observe significant differences in either the CMV-seropositive or CMV-seronegative healthy subjects.

We then performed a ROC analysis to determine the discriminatory power of CMV-specific CD4+ and CD8+ T-cell proliferation to  identify CMV+ QF+ vs. CMV+ QF− individuals. The area under the curve (AUC) yielded poor results for both CD4+ (AUC: 0.68, 95% CI 0.56–0.80; *p* = 0.038) and CD8+ T cells (AUC: 0.67, 95% CI 0.54–0.80; *p* = 0.060). These observations show that, in spite of being statistically significant, the AUC under 0.70 for CD4+ T cells demonstrated a poor discriminatory power.

### Characterization of discordant versus non-discordant CMV-seropositive subjects (CMV+ QF− vs. CMV+ QF+)

Given that the most marked differences were observed within CMV-seropositive individuals and they comprised the largest group, we further characterized these heathy participants by comparing CMV+ QF+ and CMV+ QF− subjects.

#### Serum IgG antibodies to CMV

**Figure 3:**
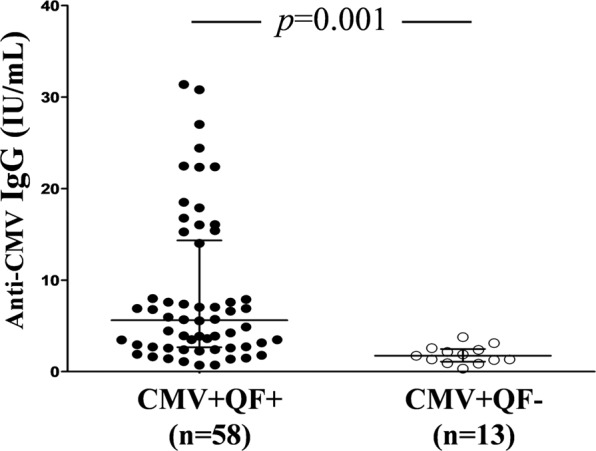
Comparative analysis of serum IgG to CMV (expressed as IU/mL) between CMV+ QF+ and CMV+ QF− individuals. Horizontal lines represent median values. The interquartile range is shown. The Mann-Whitney *U* test was used.

To investigate whether CMV+ QF+ and CMV+ QF− individuals exhibit not only proliferative but also humoral differences, we compared the level of anti-CMV IgG (IU/mL) in both groups. We found that CMV+ QF− had a significantly lower median level of IgG CMV than CMV+ QF+ subjects (1.7 IU/mL vs. 5.6 IU/mL; *p* = 0.001) (Fig. 3).

According to these results, would it be sufficient for a CMV+ individual to have a low IgGCMV level to infer a reduced CMV-specific immune response in these patients? To investigate this, we performed a ROC curve analysis to find the best IgG cut-off level to discriminate between CMV+ QF+ and CMV+ QF− individuals. We found that it had a good discriminatory power (AUC: 0.87, 95% CI 0.78–0.95; *p* < 0.001). The value with the highest sensibility (0.69) and specificity (0.92) was 3.2 IU/mL of anti-CMV IgG. The positive and negative predictive values for this cut-off were 0.97 and 0.40, respectively. The low negative predictive value indicated that only 40% of individuals with an anti-CMV IgG level lower than 3.2 IU/mL were CMV+ QF−. Therefore, a low specific IgG level was not a reliable indicator for identifying discordant individuals.

#### Combination of markers: IgG CMV level and proliferative response

**Figure 4:**
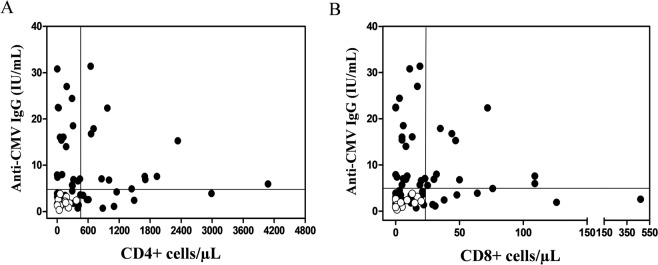
Combination of anti-CMV IgG (IU/mL) and T-cell proliferative response in discordant and non-discordant CMV−seropositive individuals (CMV+ QF− and CMV+ QF+). Solid circles (CMV+ QF+ subjects); white circles (CMV+ QF− subjects).

We then analyzed the combination of IgG CMV level (IU/mL) and proliferation capacity within CMV+ QF+ and CMV+ QF− individuals. As it is shown in Fig. 4, all the CMV+ QF− subjects had a low IgG CMV level and low lymphocyte proliferation, whereas CMV+ QF+ individuals exhibited a high heterogeneity in both parameters. Specifically, discordant CMV+ QF− individuals had an anti-CMV IgG level lower than 4 IU/mL, CD4+ lower than 400 cells/µL and CD8+ lower than 20 cells/µL.

#### Other parameters

We also analyzed whether CMV+ QF+ and CMV+ QF− showed differences in other parameters, such as sex, age or HLA alleles. Discordant CMV+ QF− subjects showed a tendency to be older than CMV+ QF+ individuals (47.9 vs. 42.7 years old; *p* = 0.087). With respect to sex, the male and females were not distributed differently between the two groups, with the frequency of females being 31.0% (18/58) in CMV+ QF+ and 38.5% (5/13) in CMV+ QF− subjects (Chi-square test *p* = 0.744). HLA allele frequencies were not significantly different between CMV+ QF+ and CMV+ QF− individuals (data not shown).

### Discordant CMV-seronegative healthy individuals (CMV− QF+)

**Figure 5:**
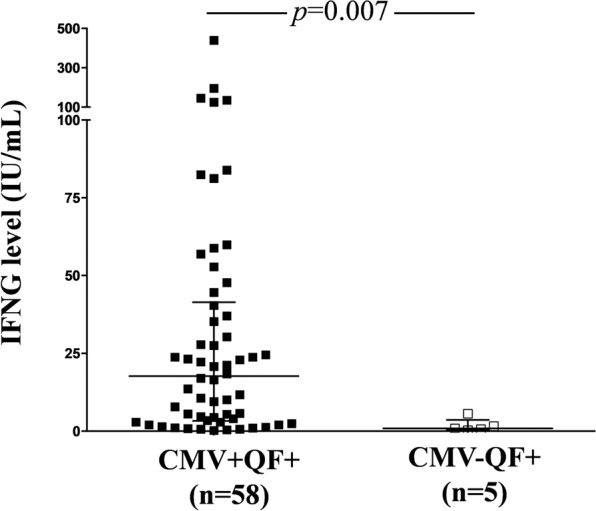
Comparison of IFNG (IU/mL) released by CMV-specific CD8+ T cells (measured by QuantiFERON-CMV assay) between CMV+ QF+ and CMV− QF+ individuals. Horizontal lines represent median values. The interquartile range is shown. The Mann-Whitney U test was used.

As reported above, we also found 5 discordant individuals within CMV-seronegative subjects (CMV− QF+). We analyzed the level of IFNG (IU/mL) to determine whether these discordant subjects had a similar level of IFNG to the CMV+ QF+ group. We found that the median IFNG release was much lower in CMV− QF+ than in CMV+ QF+ individuals (0.9 vs. 17.7 IU/mL; *p* = 0.007) (Fig. 5).

**Figure 6:**
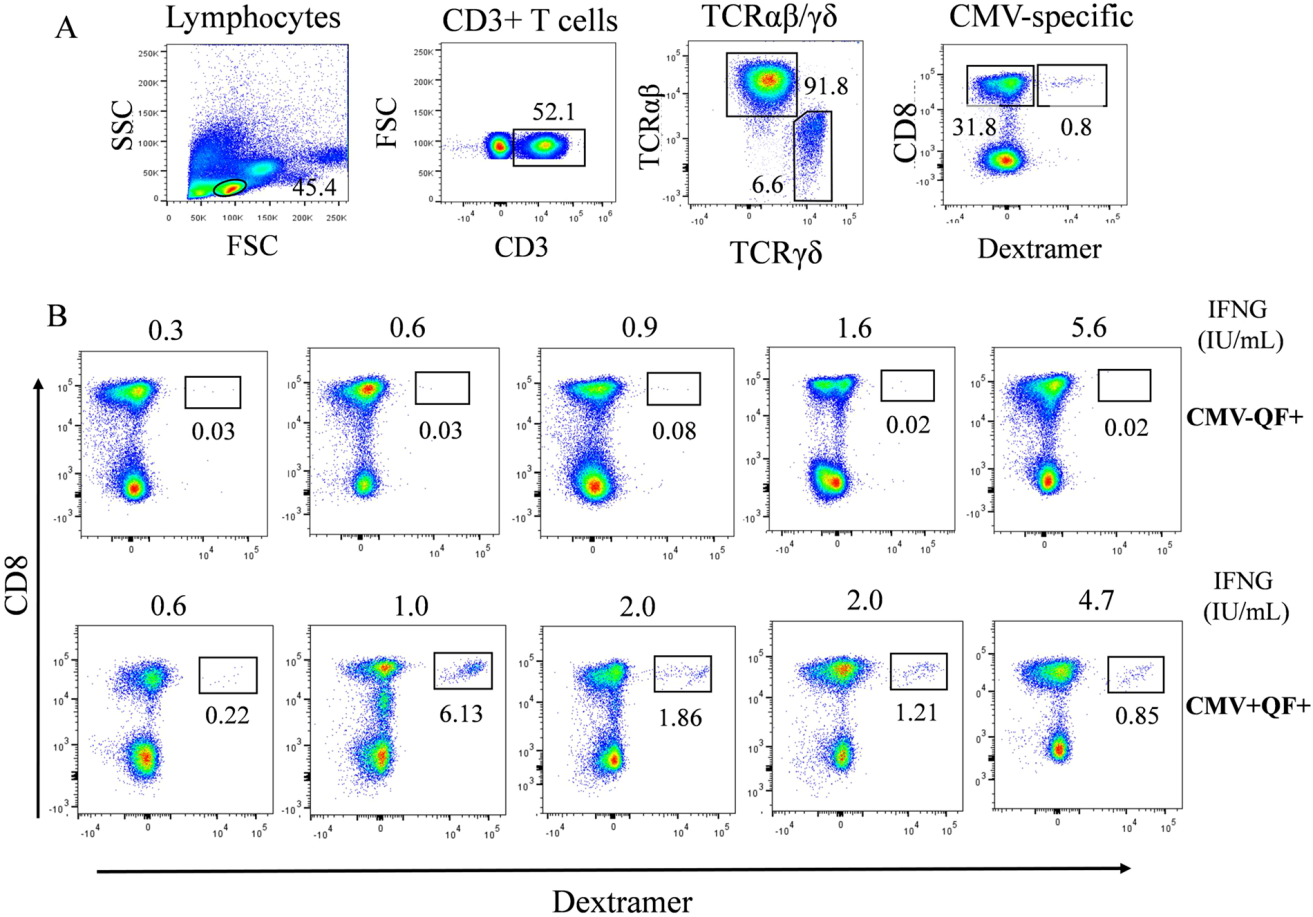
(**A**) Flow cytometry gating strategy to analyze CMV-specific CD8+ T-cell subpopulation using dextramer technology. (**B**) Quantification of this specific subpopulation in five HLA-A*02 CMV− QF+ subjects. In addition, five CMV+ QF+ individuals (4 HLA-A*02 alelle and 1 HLA-A*01) were included as the reference group. The numbers inside the panels represent the percentage of CMV-specific subpopulation with respect to total CD8+ T cells. The level of IFNG (IU/mL) for each individual measured by QF assay is shown above the panels (B).

Given that IFNG quantified by QF assay is released by CMV-specific CD8+ T cells, we analyzed this subpopulation in the discordant CMV− QF+ group. For this purpose, we used dextramer technology, which quantifies the number of CMV-specific CD8+ T cells. Since the 5 discordant CMV− QF+ individuals had the HLA-A*02 allele, we used the HLA-A*02 dextramer. As the reference group we selected 4  CMV+ QF+ HLA-A*02 subjects with a similar level of IFNG to CMV− QF+ individuals. In addition, one CMV+ QF+ subject with the HLA-A*01 allele and the lowest level of IFNG was also included in the reference group  Strikingly, none of the discordant CMV− QF+ volunteers had a detectable CMV-specific CD8+ T-cell subpopulation, whereas all CMV+ QF+ individuals, even the one with an IFNG level of 0.6 IU/mL, showed this subset (Fig. 6).

## Discussion

This study evaluates humoral/cellular CMV immunity discordance in CMV-seropositive healthy volunteers using, in parallel, two techniques that measure CMV-CMI in different ways (IFNG release vs. lymphocyte proliferation). The objective was to evaluate whether a negative QF result in CMV-seropositive individuals (CMV+ QF−) is an artifact of the QF assay, as suggested by some authors^15,17^, or it identifies individuals with immunological characteristics different from non-discordant individuals (CMV+ QF+). We found that, compared to the CMV+ QF+ individuals, the CMV+ QF− subjects are low-responder individuals since they have a reduced CMV-specific cellular response, showing a lower CD4+ and CD8+ T-cell proliferation as well as a lower anti-CMV IgG level. This indicates that a negative QF result in CMV-seropositive individuals is not associated with technical artifacts of the QF assay, but represents a well differentiated group.

When using the QF assay to measure CMV-specific T-cell response, we found that around 19% of healthy volunteers have humoral/cellular discordance (either CMV+ QF− or CMV− QF+). This is in line with findings previously reported by other authors in healthy subjects using other techniques to determine cellular immune response^10,18,19^. Discordance between serology and T-cell response has also been described in transplant patients, where it has shown to be related to the risk of CMV reactivation after transplantation^8,9,12,20^.

The difference in T-cell proliferation between CMV+ QF− and CMV+ QF+ individuals was more pronounced in CD4+ cells than in CD8+ T cells. In fact, unexpectedly, some CMV+ QF+ individuals had no CD8+ T cell proliferation with CMV lysate, which might be due to the fact that CMV lysate induces a weaker major histocompatibility class I-mediated response^19^. The discordant CMV+ QF− individuals show reduced specific immune response that is not only restricted to cellular immune response, but also to humoral immunity. Therefore, we can say that the discordance in CMV-seropositive individuals identifies a group of low responder subjects. One possible explanation for the dysfunctional response in CMV+ QF− individuals might be related to the relevant role of CD4+ T cells in the development of an appropriate antiviral immune response. It is well known that CD4+ T cells are necessary to help mount an effective specific memory CD8+ T cell response and are crucial to promote isotype switching and the production of high-affinity immunoglobulins IgG by B cells^21,22^. In this line, it has been recently published that CMV-specific CD4+ T cells and anti-CMV IgG are directly correlated^12^. Similar correlation have been reported in healthy individuals after vaccination against Hepatitis B virus^23^. Therefore, we can speculate that the impaired proliferation of CD4+ T cells with CMV lysate in CMV+ QF− subjects might influence isotype switching, thus producing a lower level of anti-CMV IgG and a lower number of CMV-specific CD8+ T cells.

In addition, discordant CMV+ QF− individuals show not only reduced specific immunity to CMV, but also against superantigen SEA + SEB. Both toxins bind in a non-specific manner to MHC class II on antigen presentation cells as well as to the T-cell antigen receptor of CD4+ T cells, producing a massive proliferation of T cells and release of proinflammatory cytokines^24,25^. Therefore, the significantly lower proliferation of CD4+ T cells with superantigen that we observed in CMV+ QF− individuals would support the role of CD4+ T cells in the discordance in CMV-seropositive individuals.

However, we also found some discordant individuals in the CMV-seronegative group since five individuals had a positive QF result, in spite of lacking IgG CMV. We cannot rule out the possibility of false negative anti-CMV IgG test results, as reported by Sester *et al*.^18^, since the serological analyses in CMV- individuals were not repeated. However, strikingly, none of them had CD4+ or CD8+ T-cell proliferation with CMV lysate or detectable CMV-specific CD8+ T-cell subpopulation with dextramer. Therefore, where does the IFNG quantified by the QF assay come from? According to Sester *et al*.^18^, one explanation might be related to an acute CMV infection in these CMV− QF+ subjects. However, the level of anti-CMV IgM was negative. Another option could be that cells other than CD8+ T cells (i.e., CMV-specific Th1 CD4+ T cells) can produce IFNG in the QF assay, since it has been reported that the peptide length distinction is not absolute and CD4+ T cells can be stimulated by antigen presented on HLA class I molecules^26^. Other alternative explanations could be related to the cross-reactivity with T cells specific for other herpes viruses or unrelated viruses^27,28^ or even that the 24 h stimulation of the QF assay led to the *in vitro* priming of CD8+ T cells^19^.

Our study has some limitations. First, in spite of our results, we cannot rule out the possibility that the negative QF result in the CMV+ QF− individuals is due to the fact that these individuals recognize other CMV antigens not represented by the peptides included in QuantiFERON-CMV. However, this is unlikely since, according to the manufacturer of the QF assay, the HLA-restricted CMV peptides of the test cover more than 98% of the population. Second, CMV lysate stimulation may provide more reliable information regarding CD4+ cells than CD8+ T cells and might not be the most suitable antigen to compare the CD8+ T-cell response of the QF assay. However, the relevant point is the significantly lower CD4+ T-cell proliferation we found in CMV+ QF− than in CMV+ QF+ individuals. Third, CMV− QF+ individuals might represent some false negative IgG test results due to the low sensitivity of some commercial IgG tests. However, in spite of this limitation, determination of serological status is the gold standard in clinical routine to classify solid organ and stem cell transplant patients according to the risk of CMV infection^3,4^.

In summary, healthy CMV+ QF− volunteers show a lower CMV-specific immunity in comparison to CMV+ QF+ individuals, either in humoral or cellular specific immunity. This discordance shows the high heterogeneity of immune response to CMV in healthy subjects. This natural heterogeneity might have clinical consequences if immunocompromised transplant patients and hematopoietic stem cell donors present this immune discordance, for whom these immunological techniques provide useful information. In this regard, interventional studies and clinical trials have demonstrated the utility of the QF assay in transplant patients, where it helps to personalize therapy against CMV after transplantation^29,30^. In addition, this study also demonstrates that a negative QF result in CMV-seropositive individuals is not an artifact but a reality.

## Materials and Methods

### Donor characteristics and study design

This cross-sectional study was carried out with healthy volunteers from the Blood Transfusion Center and Tissue and Cells Establishment of Cordoba. The participants were recruited from October 2017 to June 2019. A single blood sample was taken from each donor and CMV-serostatus and CMV-CMI response (using the QF and FASCIA assays) was determined. Informed consent was obtained from each of the subjects and the Ethics Committee of the Reina Sofia Hospital approved the study. This study was conducted in accordance with the Declaration of Helsinki.

### Determination of anti-CMV IgG and IgM antibodies

Anti-CMV IgG antibodies were determined by two commercially available methods: a semiquantitative assay (expressed as arbitrary units; AU/mL) and a quantitative assay (expressed as international units; IU/mL). In the semiquantitative assay, antibodies were analyzed by chemoluminescence at the Microbiology Unit of the Reina Sofia Hospital (Diasorin SA, Madrid, Spain). The samples with an anti-CMV IgG concentration lower than 12.0 AU/mL were considered CMV-seronegative. In the quantitative method (DIAsource Immunoassays SA, Louvain-la-Neuve, Belgium), a calibration curve and the World Health Organization international standard was used. This analysis was carried out in our lab at the IMIBIC research center.

In discordant volunteers in the CMV-seronegative group, a semiquantitative determination of anti-CMV IgM was also performed.

### HLA Class I typing

Genomic DNA was extracted from 200 μL of blood using the commercial QIAamp DNA Blood Mini Kit (QIAgen Hilden, Germany) and automated procedure (QIAcube QIAgen Hilden, Germany). HLA typing was performed using INNO-LIPA HLA-A Multiplex, which is a PCR-SSO reverse method (Fujirebio Europe N.V., Gante, Belgium). HLA-A alleles were determined with LIRASTM software for INNO-LIPA HLA. The SSP technique was used on samples that failed to be analyzed by SSO. In these cases, HLA-A locus High Res SSP Unitray Kits were used (Invitrogen by the Life Technologies Corporation, Brown Dee, Wi, USA).

### QuantiFERON-CMV assay

The QuantiFERON-CMV (QF) assay was performed according to the manufacturer’s instructions (Cellestis, a QIAGEN company, Melbourne, Australia). In brief, 1 mL of heparinized whole blood was collected in 3 QF blood collection tubes. The tubes contained either (i) a mix of 22 CMV peptides; (ii) a negative control (no antigens); or (iii) a positive mitogen control (containing phytohemagglutinin)^31^. All individuals included in the study had HLA class I alleles capable of binding CMV peptides. After collection, the tubes were shaken vigorously and incubated for 16–24 h at 37 °C. Supernatants were subsequently harvested and analyzed for IFNG (IU/mL) by standard ELISA. A result for the CMV antigen was “Positive” when the CMV antigen response minus the negative control response was higher than 0.2 IU/mL of IFNG. According to the manufacturer’s instructions, a result was “Indeterminate” when the IFNG level in the CMV antigen tube minus the negative control was less than 0.2 IU/mL and the IFNG level in the mitogen tube (once the negative control was subtracted) was less than 0.5 IU/mL.

### FASCIA assay

The FASCIA assay was performed as previously reported^32^. In the assay, whole blood was diluted at 1:9 in RPMI supplemented with L-glutamine, penicillin and streptomycin. The blood/medium mixture was stimulated with CMV viral lysate (5 μg/mL) (Microbix Biosystems Inc, Canada), Pokeweed mitogen (PWM) (5 μg/mL) (Sigma-Aldrich, USA), *Staphylococcus aureus* enterotoxins A and B (SEA + SEB) (0.1 μg/mL of each) (Sigma-Aldrich, USA) or RPMI (unstimulated control) in sterile polypropylene Falcon 12 ×75 mm tubes to a final volume of 500 μL. Pokeweed mitogen (PWM) is a lectin from *Phytolacca americana* that promotes polyclonal differentiation of B cells. The *staphylococcal* enterotoxins A and B are considered superantigens that stimulate large populations of T cells. The tubes were incubated for 7 days at 37 °C, 5% CO_2_ and 95% humidity. After this time, the supernatant was collected and stored at −80 °C. The cells were subsequently stained with anti-CD3 AF700 (BD Biosciences, USA) and anti-CD8 APC (Miltenyi Biotec, Germany). In addition, the medium tube and PWM tube were stained with anti-CD19 Viogreen (Miltenyi Biotec, Germany). All the tubes were incubated for 10 minutes in the dark at room temperature. The erythrocytes were then lysed with Test lysis buffer (Beckman Coulter, USA) and the samples were centrifuged at 1800 rpm for 5 minutes. The supernatant was discarded and the cells were resuspended in 450 μL of phosphate buffered saline with 1% of bovine serum albumin (PBSA). Blast numbers were acquired for 80 seconds in an LSR Fortessa flow cytometer (Beckman Coulter, CA, USA).

The absolute number of proliferating cells was calculated using a Trucount tube (BD Biosciences, CA, USA), as previously reported^32^. The values of the unstimulated samples (with RPMI) were subtracted from the values of the stimulated samples.

### Quantification of CMV-specific CD8+ T-cell subpopulation with dextramers

The number of CMV-specific CD8+ T cells was quantified using dextramer technology (Immudex, Denmark) and performed according to the manufacturer´s recommendations. HLA-A2-restricted NLVPMVATV (pp65) and HLA-A1-restricted VTEHDTLLY (pp65) dextramers were used. Approximately 0.5×10^6^ PBMCs were incubated with specific dextramers (5 µL) for 10 minutes at room temperature. The cells were then stained with anti-CD3 AF700 (BD Biosciences), TCR αβ (APC), TCR γδ (APC-Vio700) and anti-CD8 PE-700 (Miltenyi Biotec) and incubated for 15 min at 4 °C in the dark. After two washes with PBSA, the cells were acquired in an LSR Fortessa flow cytometer (Becton Dickinson). Post-acquisition analysis was performed using FlowJo software version vX.0.6 (Tree Star, USA). Dot plots were generated using FlowJo vX.0.6 software.

### Statistical analysis

The statistical analysis was performed using IBM SPSS Statistics 24.0 software (SPSS Inc., Chicago IL, USA). Categorical variables were compared using the Chi-square or Fisher tests. Comparison of quantitative variables was assessed using the Mann-Whitney *U* test. Values were considered statistically significant when the *p*-value was <0.05. Graphic presentation was performed with GraphPad Prism 7 (GraphPad Sofware Inc).

## Data Availability

The datasets generated during and/or analysed during the current study are available from the corresponding author on reasonable request.
